# Low grade papillary transitional cell carcinoma pelvic recurrence masquerading as high grade invasive carcinoma, ten years after radical cystectomy

**DOI:** 10.1186/1477-7819-6-103

**Published:** 2008-09-30

**Authors:** Pankaj P Dangle, Wenle Paul Wang, Joel Mayerson, Amir Mortazavi, Paul Monk

**Affiliations:** 1 The James Cancer Hospital and Solove Research Institute, Ohio State University and Comprehensive Cancer Center, Columbus Ohio, 43210, USA; 2 Department of Pathology, The Ohio State University, Columbus Ohio, 43210, USA; 3 Department of Orthopedics, The Ohio State University, Columbus Ohio, 43210, USA; 4 Department of Hematology and Oncology, The Ohio State University, Columbus Ohio, 43210, USA

## Abstract

**Background:**

Tumor recurrence following radical cystectomy for a low-grade superficial transitional cell carcinoma (TCC) is exceedingly uncommon and has not been reported previously.

**Case presentation:**

We describe a case of a young male presenting with anorexia, weight loss and a large, painful locally destructive pelvic recurrence, ten years after radical cystoprostatectomy. The pathology was consistent with a low-grade urothelial carcinoma. After an unsuccessful treatment with cisplatin-based chemotherapy, the patient underwent a curative intent hemipelvectomy with complete excision of tumor and is disease free at one year follow-up.

**Conclusion:**

A literature review related to this unusual presentation is reported and a surgical solutions over chemotherapy and radiotherapy is proposed.

## Background

Low-grade papillary (Ta) urothelial carcinomas have the lowest risk of progression to invasive disease and death of all the superficial tumor types, with 50–70% recurrence rate after transurethral resection of bladder tumor (TURBT) and progression to invasive disease in 2.4–3.3% of cases [1]. In comparison, the high-grade disease managed with TURBT alone recurs in 80% of cases and becomes invasive in 50% [2]. We describe an unusual case of an aggressive low-grade papillary urothelial carcinoma recurrence ten years following radical cystectomy.

## Case presentation

A 48 year old male with a long history of smoking presented with weight loss, anorexia and pelvic pain. He had a significant past history of a radical cystectomy ten years prior for a large multi-focal non-invasive, low-grade papillary (Ta) transitional cell carcinoma. The stated indications for cystectomy were large size of the mass and the anticipated inability to perform a complete resection. The pathological specimen which was reviewed at our institution was described as a low-grade non invasive papillary multifocal transitional cell carcinoma (TCC). The margins were clear and fourteen uninvolved lymph nodes were submitted. Postoperatively the patient recovered well and was under surveillance without any disease till above mentioned complaint. The patient's past history was also significant for a straddle injury requiring open surgical repair that occurred approximately 2 years prior to the diagnosis of bladder cancer.

**Figure 1:**
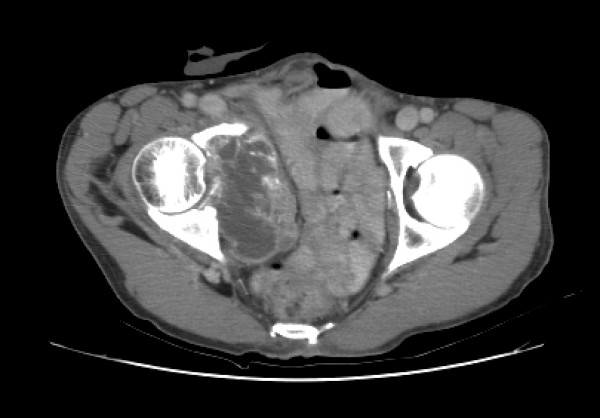
**CT scan of pelvis showing a large locally destructive mass lesion**. Showing a right sided large heterogeneous pelvic mass with an area of central necrosis with evidence of bone destruction (right acetabular invasion) and distal rectal involvement.

Physical examination revealed a thin uncomfortable male with no other abnormal findings. Basic laboratory investigations were within normal limits. Imaging studies with CT scan of abdomen and pelvis revealed a right sided large heterogeneous pelvic mass with an area of central necrosis and evidence of bone destruction (right acetabular invasion) and distal rectal involvement (Figure 1). There was no evidence of disease spread beyond this destructive pelvic mass.

A CT guided biopsy of this mass revealed a low-grade urothelial carcinoma. Cisplatin based chemotherapy along with growth factor support was administered [dose dense methotrexate, vinblastine, doxorubicin and cisplatin (MVAC)]. After 3 uncomplicated cycles no tumor response was achieved. It was then decided that a curative intent *en bloc *resection represented the best option for patient.

**Figure 2:**
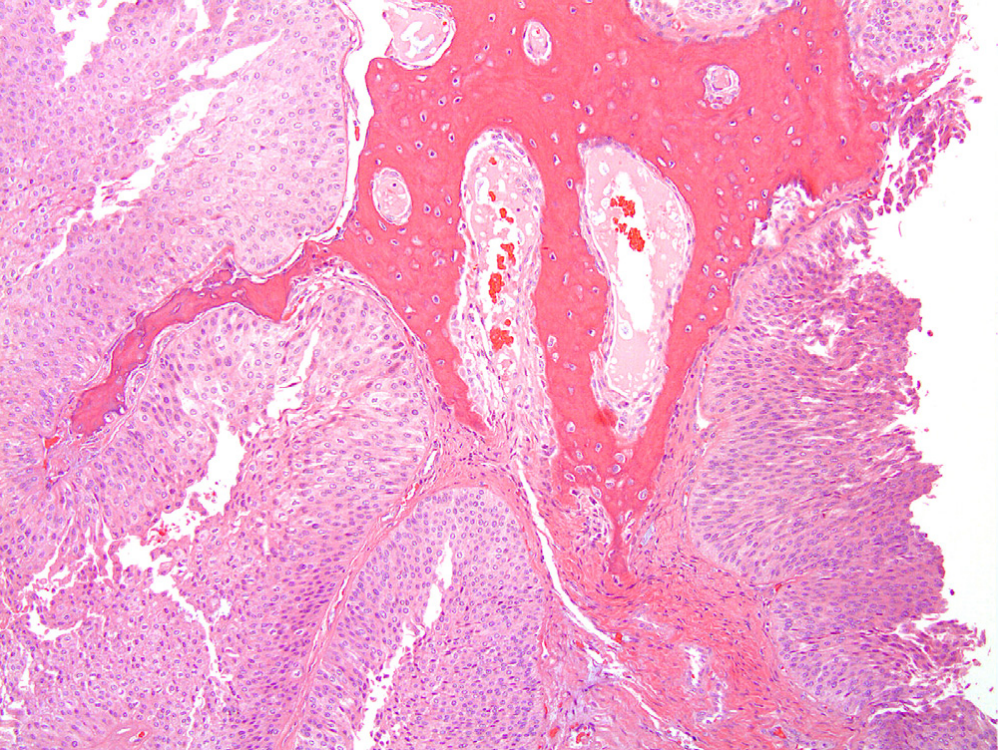
**Low grade papillary urothelial carcinoma infiltrating pelvic bone.** At low magnification (10 × 10) the low grade urothelial carcinoma forms nests and infiltrates cortical bone.

The patient underwent surgical resection of the mass requiring a right hemipelvectomy, end colostomy and a myocutaneous flap closure with penile and scrotal reconstruction. The final pathology revealed an urothelial cell tumor with predominantly low-grade morphologic features, with focal areas of high grade tumor seen (Figure 2; low magnification 10 × 10). The tumor invaded bone and soft tissue in a broad-based pushing fashion. The tumor formed nests with infiltration in the cortical bone, dissecting the pelvic soft tissue. There was no lymphovascular invasion and surgical margins were not involved. The patient is free from disease recurrence after more than one year following surgery.

## Discussion

Risk factors for urothelial carcinoma recurrence after cystectomy have been identified. Tumor grade (G), extent of invasion (T) and lymph node involvement (N) are the most widely recognized, beside others [3]. Herr *et al*., in a multivariate analysis of 268 patients suggested that apart from pathologic and nodal stage, number of lymph nodes removed also influences the local recurrence and the disease specific survival [4]. Data regarding risks of recurrence is limited to intermediate and high-grade disease and for the most part diseases that are considered invasive, which highlight the rarity of the presented case. Five-year survival for high-grade Ta disease following radical cystectomy is between 88–100% [5]. The same statistics for low-grade disease have not been reported, but is expected to be far better.

Various site of metastasis such as skin, lung, orbit metatarsal bone, penis, posas muscle and calcaneum have been reported in the literature in patients with superficial bladder cancer [6-9].

Saito reported a case of solitary subcutaneous scrotal metastasis 18 months following initial treatment with TURBT and intravesical instillation of Bacillus Calmette-Guérin (BCG) with no tumor recurrence on repeat cystoscopy. The histology of scrotal lesion was consistent with the primary bladder tumor showing intermediate grade transitional cell carcinoma (pT1a) disease [6].

Ku *et al*., reported a case of delayed recurrence 20 years following radical cystectomy for a low-grade muscle invasive disease with skin and pelvic metastasis. The histology from skin recurrence was consistent with well-differentiated TCC. Subsequently patient developed a pelvic recurrence in spite of chemotherapy [10]. In our experience too the patient failed to respond to the cisplatin based chemotherapy as reported in above mentioned study. Though, this patient and our case had the same grade of disease, interestingly, this patient had an invasive (pT2 N0 M0) disease comparing to our case who had a non-invasive (pTa N0 M0) disease.

Kumar *et al*., reported a case of vaginal and omental metastasis six years after TURBT for a well-differentiated superficial TCC. Subsequent evaluation revealed no visible tumor in the bladder, but large omental deposit and left obturator lymph node mass engulfing the ureter. The report does not document the grade of recurrent TCC [11].

Recently Dougherty *et al*. [12], reported two cases of lung metastasis in patients with low-grade superficial bladder cancer. Both patients presented with lung metastasis with an underlying low-grade disease in bladder. Both patients underwent metastatectomy, and platinum-based chemotherapy with a partial response. Neither patient underwent a cystectomy for the primary disease [12].

There are many similarities of the above cases in the literature to our case. To our knowledge our case is the first reported case of a non-invasive low grade urothelial carcinoma treated with cystectomy with a late recurrence of the same low-grade disease. The value of the cystectomy in our case is high, because of the well known problem of clinical understaging in urothelial carcinomas (Table 1).

**Table 1:** Published case reports involving low grade TCC distant metastasis following either bladder preserving techniques or radical cystectomy.

**Author**	**Bladder Histology**	**Primary treatment**	**Duration of recurrence**	**Site of Recurrence**	**Histology of recurrence**
Saito (1998) [6]	Intermediate	TURBT and BCG	18 month	Scrotal skin	Intermediate
Kumar et al (2001) [11]	Well differentiated	TURBT	6 years	Omental, Left pelvic lymph node mass	N/A
Ku etal (2005) [10]	Low grade Invasive	Radical Cystectomy	20 years	Skin and Pelvis	Well Differentiated
Dougherty et al (2008) [12]	Low Grade Sup. TCC	Multiple TURBT's and Intravesical therapy	Case 1–10 years Case 2–15 years	Lung metastasis	Low grade

The mechanism responsible for such a delayed presentation in our case is unknown. It is very likely that the tumor was seeded in the pelvic area over 10 years prior, and considering the location of the tumor and its low-grade, it did not become symptomatic for many years. The history of saddle injury and/or the repair of this injury may have played a role in this case. Traumatic implantation of the cancer cell is supported by a report of similar implantation metastasis following laparoscopic bladder biopsy for bladder cancer [13]. Thus a proposed possibility could be linked to the precedent traumatic urethral injury with local extravasation and possible implantation.

Modern cisplatin-based combination chemotherapy regimens are associated with 40–60% objective response rates in metastatic high-grade urothelial carcinomas. The regimen used in our case is associated with an overall response rate of 62% [14]. Our intent was to shrink the patient's tumor to enable a smaller surgery. The lack of tumor response however is not surprising given the tumor's low-grade and likely low mitotic rate.

## Conclusion

We present an exceedingly rare occurrence of a pelvic recurrence of a low-grade superficial TCC after cystectomy. Delayed presentation with recurrent low-grade urothelial carcinoma is an unusual entity and potential mechanism of traumatic implantation should be considered. Characteristically low-grade tumor's are resistant to systemic chemotherapy and curative-intent surgical resection of the tumor should be considered.

## List of abbreviations

TURBT: Transurethral resection of bladder tumor; TCC: Transitional cell carcinoma; MVAC: Methotrexate, vinblastine, doxorubicin and cisplatin.

## Competing interests

The authors declare that they have no competing interests.

## Authors' contributions

PPD – concept and design, collection and assembly of data, analysis and interpretation of data and preparation of manuscript. WPW – provided study material and patient, editing of the manuscript and approval of final draft. JM – provided study material and patient, editing of the manuscript and approval of final draft. AM – provided study material and patient, editing of the manuscript and approval of final draft. PM – Conception and design, provided study material and patient, data analysis and interpretation and preparation and editing of manuscript. All authors read and approved the final manuscript.

## Consent

Written informed consent was obtained from the patients for publication of this case report and any accompanying images. A copy of written consent is available for review by the Editor-in-Chief of this journal.
